# Smart Decentralization of Personal Health Records with Physician Apps and Helper Agents on Blockchain: Platform Design and Implementation Study

**DOI:** 10.2196/26230

**Published:** 2021-06-07

**Authors:** Hyeong-Joon Kim, Hye Hyeon Kim, Hosuk Ku, Kyung Don Yoo, Suehyun Lee, Ji In Park, Hyo Jin Kim, Kyeongmin Kim, Moon Kyung Chung, Kye Hwa Lee, Ju Han Kim

**Affiliations:** 1 Division of Biomedical Informatics College of Medicine Seoul National University Seoul Republic of Korea; 2 Division of Nephrology Department of Internal Medicine Inje University Seoul Paik Hospital Seoul Republic of Korea; 3 Division of Nephrology Department of Internal Medicine Ulsan University Hospital Ulsan Republic of Korea; 4 Department of Biomedical Informatics College of Medicine Konyang University Daejeon Republic of Korea; 5 Department of Internal Medicine Kangwon National University Hospital Chuncheon Republic of Korea; 6 School of Medicine Kangwon National University Chuncheon Republic of Korea; 7 Department of Internal Medicine and Biomedical Research Institute Pusan National University Hospital Busan Republic of Korea; 8 Department of Internal Medicine Eulji University Hospital Daejeon Republic of Korea; 9 College of Medicine Eulji University Daejeon Republic of Korea; 10 Department of Information Medicine Asan Medical Center Seoul Republic of Korea; 11 College of Medicine University of Ulsan Seoul Republic of Korea

**Keywords:** personal health records, blockchain, mobile health, semantic interoperatbility, decentralized system, patient-centered system

## Abstract

**Background:**

The Health Avatar Platform provides a mobile health environment with interconnected patient Avatars, physician apps, and intelligent agents (termed *IoA^3^*) for data privacy and participatory medicine; however, its fully decentralized architecture has come at the expense of decentralized data management and data provenance.

**Objective:**

The introduction of blockchain and smart contract technologies to the legacy Health Avatar Platform with a clinical metadata registry remarkably strengthens decentralized health data integrity and immutable transaction traceability at the corresponding data-element level in a privacy-preserving fashion. A crypto-economy ecosystem was built to facilitate secure and traceable exchanges of sensitive health data.

**Methods:**

The Health Avatar Platform decentralizes patient data in appropriate locations (ie, on patients’ smartphones and on physicians’ smart devices). We implemented an Ethereum-based hash chain for all transactions and smart contract–based processes to guarantee decentralized data integrity and to generate block data containing transaction metadata on-chain. Parameters of all types of data communications were enumerated and incorporated into 3 smart contracts, in this case, a health data transaction manager, a transaction status manager, and an application programming interface transaction manager. The actual decentralized health data are managed in an off-chain manner on appropriate smart devices and authenticated by hashed metadata on-chain.

**Results:**

Metadata of each data transaction are captured in a Health Avatar Platform blockchain node by the smart contracts. We provide workflow diagrams each of the 3 use cases of data push (from a physician app or an intelligent agents to a patient Avatar), data pull (request to a patient Avatar by other entities), and data backup transactions. Each transaction can be finely managed at the corresponding data-element level rather than at the resource or document levels. Hash-chained metadata support data element–level verification of data integrity in subsequent transactions. Smart contracts can incentivize transactions for data sharing and intelligent digital health care services.

**Conclusions:**

Health Avatar Platform and interconnected patient Avatars, physician apps, and intelligent agents provide a decentralized blockchain ecosystem for health data that enables trusted and finely tuned data sharing and facilitates health value-creating transactions with smart contracts.

## Introduction

Personal health records are an electronic health information resource derived from multiple data sources that are integrated, managed, and controlled by individuals [1,2]. Through the use of a personal health record, a person can not only gain more knowledge about their current health conditions but can also receive assistance when seeking an appropriate treatment plan [3,4]. To maximize the utilization of personal health record, it is necessary to develop a system that allows patients to access, manage, and exchange their health information easily and safely and apply appropriate standards [5,6]. Recently, with the progress of Internet of Things (IoT) technology, patients can obtain various types of health-related information through a range of mobile devices or sensors [7]. The integrity of a personal health record demands both syntactic and semantic interoperability and the patient-centered health-data integration of various sources, including institutional electronic health records, IoT-enabled life logs, patient-reported outcome measures, and personal genomic data.

There is a need for an electronic environment for patient personal health records to interact with both physician apps and third-party artificial intelligence service agents. The Health Avatar Platform (HAP) began as decentralized health data management platform supporting patient-centered health data integration on a mobile smartphone app (a patient Avatar). HAP allows patients to store and manage their health data received from various health care institutions with syntactic and semantic interoperability. Once authorized and registered, third-party agents or distributed artificial intelligence services can access patient-centric health data on patient Avatars through HAP RESTful (representational state transfer) application programming interfaces (APIs) [8,9]. Electronic health record data can be pushed to an institutional gateway server (XNetHub) and then pulled by physician apps (XNet). Syntactic interoperability is supported by standard messaging protocols such as HL7 FHIR (Fast Healthcare Interoperability Resources), HL7 Continuity of Care Document, and ASTM Continuity of Care Record protocols. Semantic interoperability of electronic health record data from different hospitals is secured by predefined, preregistered, and postexpandable common data elements that full comply with ISO/IEC 11179 metadata registry international standards [8].

HAP enables peer-to-peer bidirectional communications among patient Avatars, third-party intelligent agents, and physician apps (termed *IoA^3^*). DialysisNet (a physician app) and Avatar Beans (a patient Avatar) were the first and most successful apps for chronic kidney disease and hemodialysis patient management. The Avatar Beans app is downloadable from Google Play [9] and Apple App Store [10]. As of December 1, 2020, these apps were used by 22 nephrologists connecting 14 teaching hospitals using different electronic health records and 245 patients in South Korea. Kim et al [11] successfully conducted a multicenter cohort study for evaluating the treatment patterns of renal anemia of patients undergoing hemodialysis using DialysisNet. DialysisNet demonstrated seamless connectivity and semantic interoperability of standardized electronic clinical data capture among heterogeneous electronic health record systems. RehabilitationNet and Avatar Fit were launched (in 2020) as a second wave for an industrial-accident hospital network.

As public ledger technology [12,13], blockchain records transaction data between participants on a network in tamper-resistant storage [14]. Smart contracts can be developed and deployed by Ethereum [15,16], allowing contracts or interactions between participants in the blockchain network to be represented in Turing-complete language and automatically executed [17]. Many medical institutions and health care vendors have been conducting research on blockchain methods to build a system that is more transparent, traceable, verifiable, and irreversible with transactions occurring in conventional information systems [18-24]. The main objective of most of these studies was to share and exchange medical information mainly generated by providers securely. Others have applied a blockchain network for personal health record management [23,25-31]; these studies focused on a mobile health platform for patients to manage patient-centered health information.

Serving solely as an intermediary, HAP does not store any health data but securely relays authenticated and authorized data transmissions in a fully decentralized fashion among mobile devices and servers of interconnected patient Avatars, physician apps, and intelligent agents. In other words, even before the introduction of blockchain, HAP has already been a fully decentralized blockchain-friendly electronic or personal health record management platform. HAP is not vendor- or provider-centric but patient-centric. Because HAP is a mobile device–based health data integration or exchange platform for patients (ie, Avatars) and physicians (ie, apps) with no central storage, there are no privacy risks (eg, unauthorized access) as there are in centralized management systems [32-34]. HAP supports iPads for physician apps for security reasons and both iOS and Android smartphones for patient Avatars for broader acceptance. Therefore, the introduction of blockchain technology to HAP and interconnected patient Avatars, physician apps, and intelligent agents systems may be a natural evolutionary path for better decentralization of digital health care. It resolves the old problems of (1) managing redundancy and integrity in decentralized health data among interconnected patient Avatars, physician apps, and intelligent agents devices; (2) verifying data authenticity and provenance; and (3) protecting data security from interference, forgery, and tampering by means of immutability. Blockchain technology guarantees better trust with regard to these functionalities [14]. Furthermore, the introduction of smart contract technology enables (4) smart access control down to each data element level from the current document or resource level, (5) smart data sharing at each data element level, and (6) a crypto-economy ecosystem for correctly incentivizing health care behaviors while also ensuring data privacy protection.

This paper describes (1) HAP and interconnected patient Avatars, physician apps, and intelligent agents system architecture for decentralized health data management by means of hash chain, RESTful API, and smart contract–based processes of authorized (2) data pushes to patient Avatars and to physician apps by each other, (3) data pulls from Avatars and apps upon a request from an intelligent agent for the purpose of decision support, and (4) data backup into a secure backup storage. A physician can prescribe a scheduled questionnaire to a patient and collect patient-reported outcome measures by combining these processes. Moreover, while standard messaging protocols such as HL7 FHIR, and HL7 Continuity of Care Document or ASTM Continuity of Care Record allow resource-level bulk queries, each common data element–level detailed query for data push and pull instances is supported by HAP interconnected patient Avatars, physician apps, and intelligent agents implementations by means of smart contracts. Each step in the processes can be systematically incentivized by the crypto-economy to facilitate data transactions and healthy behaviors in the HAP interconnected patient Avatars, physician apps, and intelligent agents ecosystem.

## Methods

### Health Avatar Platform Architecture and Data Communication

**Figure 1:**
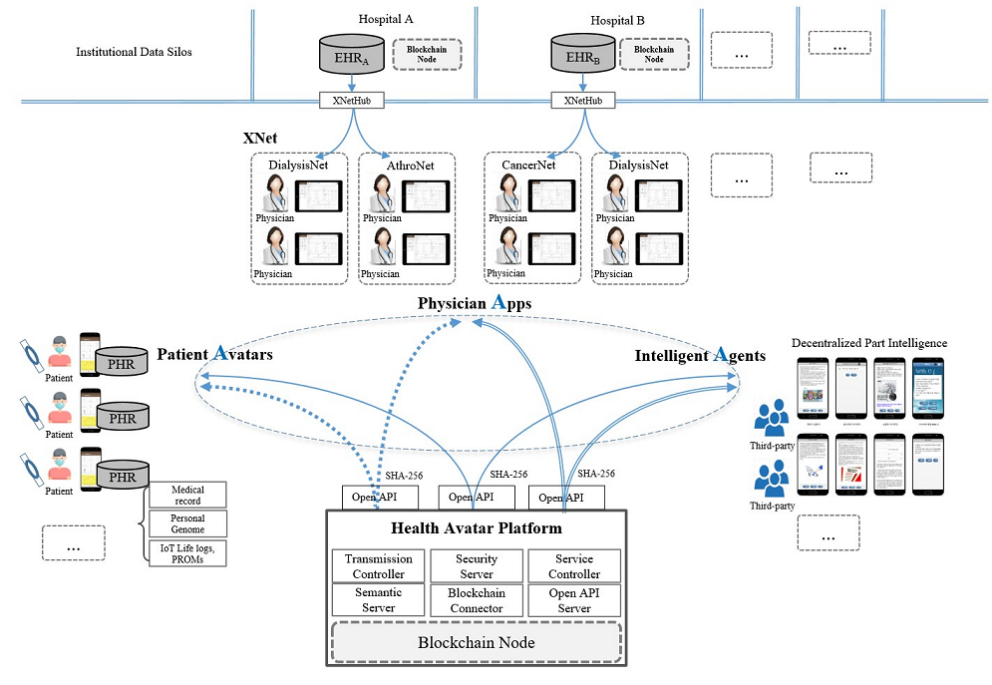
*IoA^3^* (internet of Avatars, apps, and agents) system architecture. All data communications between entities are authenticated and hash-audited by the Health Avatar Platform to ensure data provenance. Blockchain nodes are distributed among the participating hospitals. API: application programming interface; EHR: electronic health record; PHR: personal health record; PROM: patient-reported outcome measure.

Avatars, apps, and agents of interconnected patient Avatars, physician apps, and intelligent agents represent patients, physicians, and third-party digital health care service providers, respectively. HAP has no central health data storage and performs decentralized data management (Figure 1). Patient data from many electronic health record systems are extracted, transformed via metadata validations, loaded onto a gateway XNetHub, and then synchronized with physician apps. Relevant personal health data are pushed from physician apps to patient Avatars, permitting an institutional data policy to be implemented. Smartphone-enabled patient Avatars are at the center of patient-centered data integration by collecting and integrating fragmented health data from multiple health care institutions. Patients can also generate patient-reported outcome measures that can be stored and sent to appropriate physician apps. Intelligent agents can request that physician apps or patient Avatars transmit health data via HAP RESTful APIs to provide digital health care services that are certified by and registered to the platform. Agents’ recommendations and analysis results can also be sent to Avatars and XNets. The HAP server authenticates and authorizes instances of data access and transmission by Avatars, apps, and agents (or interconnected patient Avatars, physician apps, and intelligent agents) via smart contracts.

Fully decentralized health data management enabling strong data privacy is the hallmark of the HAP and interconnected patient avatar, physician app, and intelligent agent system. Each data element resides in its proper location (ie, a patient’s data in the corresponding patient Avatar, a physician’s data in the physician app, and a service provider’s data in a third-party agent). Data redundancy is inevitable when a patient sends a copy of their patient-reported outcome measure to a physician and when a physician sends a copy of electronic health record data such as laboratory results and medications, to a patient. Intelligent agents can also receive health data and send (expert system) recommendations for clinical decision support to physicians as well as directly to patients. Previously, provenances of redundant decentralized health data were managed by a legacy HAP system. Introducing a hash chain for each data transaction among interconnected patient Avatar, physician app, and intelligent agent entities ensures better data provenance.

HAP provides a mobile platform for highly interconnected personal health records connecting many health care institutions, patients, and decentralized artificial intelligence agents. Semantic interoperability during data exchanges is achieved by fully curated and registered clinical common data elements supporting the ISO/IEC 11179 Metadata Registry standard. Electronic health record data are automatically transformed into common data elements at the time of extraction, transformation, and loading into the XNetHub metadata registry server. The metadata registry documents the standardization and registration of metadata to make the data understandable and shareable. HL7 FHIR, HL7 Continuity of Care Document, and ASTM Continuity of Care Record standards are supported (Multimedia Appendix 1) and semantically enriched at each common data element level by the metadata registry server for each of the specific apps. HAP has been used by 2 real-world practices in Korea—DialysisNet for chronic kidney disease and RehabilitationNet for neuromusculoskeletal disability management.

### Application of a Blockchain Network

Though HAP has already been used for decentralized health data management in clinical practices, it is challenging for the legacy HAP system to verify whether or not the data on a terminal device such as a patient’s smartphone have been compromised. We implemented an Ethereum-based hash chain as a tamper-proof and traceable modular storage approach to guarantee data integrity among terminal devices by storing a hash for each data transmission and applying them to verify data authenticity between originals and the copies of transmitted data. Thus, all data that have ever been transmitted through HAP can be correctly verified by HAP hash audits without risk to privacy (such as those that arise from capturing sensitive health data in a central storage). A patient’s own patient-reported outcome measures from wearable devices or self-reporting forms can be verified for data provenance when patients send these records to themselves for digital signing or to another entity through hash auditing.

Health data are stored and managed off-chain in a decentralized fashion. The platform serves as a relay server that only stores the hash values on-chain of all data transactions for verification, data provenance, and auditing for tamper-proof data privacy. Two modules, called Blockchain Monitor and Node Manager, were newly added to the legacy HAP for creating block data in Ethereum (Multimedia Appendix 2). Introducing a blockchain technology to a legacy health care information system requires careful consideration, even with a popular and technically mature solution [35]. While Bitcoin is considered to be the most secure and representative platform, Ethereum is one of the most popular and robust platforms (1) allowing smart contract to be executed on-chain, (2) providing both permissioned and permission-less blockchain network, and (3) supporting a protocol-based crypto-economy environment, which is essential in incentivizing highly regulated but heterogeneous interactions [36,37]. The Ethereum network’s proof-of-authority consensus algorithm was used [38]. Unlike a permission-less blockchain, the proof-of-authority algorithm can manage participants in the blockchain network. This algorithm also allows participants or initial nodes in the blockchain network to act as block generators for nodes that are new in the network. Because health-related information can be categorized as sensitive personal information, unauthorized access by other users should be prevented. If the permission-less blockchain approach is applied to a mobile health system, patient information is bound to be passed on to unreliable anonymous nodes. The application of permissioned blockchain is more appropriate to curb the potential for the occurrence one such breach. In this study, this permissioned blockchain was designed to allow only preconsulted care providers or health organizations to participate as nodes. Additionally, proof-of-authority Ethereum can create blocks more rapidly than permission-less blockchain networks method such as proof of work [12,39].

### Off-Chain Data Management

To capture transaction hash logs in the blockchain, smart contracts that can be executed on an Ethereum virtual machine are required. Table 1 summarizes the parameters that can be extracted from data communications for each step. We designed smart contracts based on these investigated parameters. By designing these contracts, we intended to manage transaction metadata on the blockchain for data provenance while also managing patients’ health data off-chain safely in their proper locations(ie, patient smartphones, physicians’ smart pads, and agents' servers) for strong privacy protection. In addition, it is necessary to design a process that executes additionally implemented contracts in the legacy data communication process and delivers the required parameters; therefore, we compared different blockchain architectures (Multimedia Appendix 3).

We implemented Go-Ethereum blockchain with the smart contracts (Table 1) on CentOS (version 7.2; Linux) along with initial 3 proof-of-authority blockchain nodes of DialysisNet on HAP. We set the block generation cycle of the nodes to 5 seconds. For the purpose of performance evaluation, we tested the use case scenarios using sample data sets including data elements for simulated hemodialysis patients’ vital signs, laboratory results, and medications.

**Table 1:** Parameters delivered in data transmission scenarios. Parameters are considered as metadata for transmitted health data and must be stored and managed in the blockchain.

Scenario and steps		Departure	Destination	Name of parameter	Data type	Description
**Agent or app sends data to Avatar**						
	1	Physician app	Patient Avatar	senderID	string	Unique identifier of the data sender (app)
				receiverID	string	Unique identifier of the data receiver (Avatar)
				dataSegment	JSON^a^	Sent data segment by the sender
				timestamp	datetime	Timestamp for data transmission
**Agent or app requests data to Avatar**						
	1	Agent or physician app	Patient Avatar	API^b^	string	API syntax including requests for detailed data query
				senderID	string	Unique identifier of the data sender (Avatar)
				receiverID	string	Unique identifier of the data receiver (agent or app)
				timestamp	datetime	Timestamp for data transmission
	2	Patient Avatar	Agent or physician app	dataSegment	JSON	Sent data segment by the sender.
**Agent sends data to app**						
	1	Agent	Physician app	senderID	string	Unique identifier of the data sender (agent)
				receiverID	string	Unique identifier of the data receiver (app)
				timestamp	datetime	Timestamp for data transmission
				dataSegment	JSON	Sent data segment by the sender
**Agent requests data to app**						
	1	Agent	Physician app	API	string	API syntax including requests for detailed data query
				senderID	string	Unique identifier of the data sender (app)
				receiverID	string	Unique identifier of the data receiver (agent)
				timestamp	datetime	Timestamp for data transmission
	2	Physician app	Agent	dataSegment	JSON	Sent data segment by the sender

^a^JSON: JavaScript object notation.

^b^API: application programming interface.

## Results

### Smart Contracts and Use Cases

Each Ethereum node stores and manages transaction metadata during the course of all data exchanges on the HAP interconnected patient Avatars, physician apps, and intelligent agents. SC-1, as the health data transaction manager, stores *senderAddr* (the account address of the sender of the data segment), *receiverAddr* (the account address of the receiver), *HashedDS* (the hash value of the data segment through the Secure Hash Algorithm-256 function), and *HashSeq* (a unique key for the transaction), which can also be used as a foreign key between contracts. SC-2, as the health data transaction status manager, manages the status of data transactions to be saved by SC-1. Finally, SC-3, as the HAP API transaction manager, was developed to manage information related to an agent's personal health record data requests. SC-3 manages the hash value of the requested API (Table 2).

Patient data are located in their smartphones (Avatar), physician’s data for their patients are located in their smart Pads (XNet), agent’s data for its customer are located in its server, and the health care institution’s data are located in its electronic health record or other production servers. Thus, data are primarily stored and managed off-chain. All data transmission logs to proper receivers are on-chain through the HAP hash-and-relay server with a proper rationale and at a proper time (Figure 1). Node Manager manages information about each node that makes up the blockchain network. Information on personal health record data transactions are transmitted or requested to be traced to Blockchain Monitor (Multimedia Appendix 2). Blockchain verifies personal health record data managed off-chain in HAP or stores transaction metadata for verification. All transaction can be properly incentivized.

**Table 2:** Smart contracts (SC-1, SC-2, and SC-3) and variables in each contract.

Smart contract and variable			Data type		Description
**SC-1: Health data transaction manager**					
	senderAddr	address		Address of the health data sender’s Ether account	
	receiverAddr	address		Address of the health data receiver’s Ether account	
	HashedDS	string		Hashed string value of data segment	
	HashSeq	uint256		Unique sequence for identification of the *HashedDS* value	
**SC-2: Health data transaction status manager**					
	contractAddr	address		Address of the smart contract account	
	HashSeq	uint256		Unique sequence for identification of the *HashedDS* value	
	status	string		Status of health data transaction. (eg, “waiting,” “complete”)	
**SC-3: HAP^a^ API^b^ transaction manager**					
	hashedAPI	string		Hashed string value of agent API syntax.	
	HashSeq	uint256		Unique sequence for identification of the *HashedDS* value	

^a^HAP: Health Avatar Platform.

^b^API: application programming interface.

### Push: Updating via the Physician App

**Figure 2:**
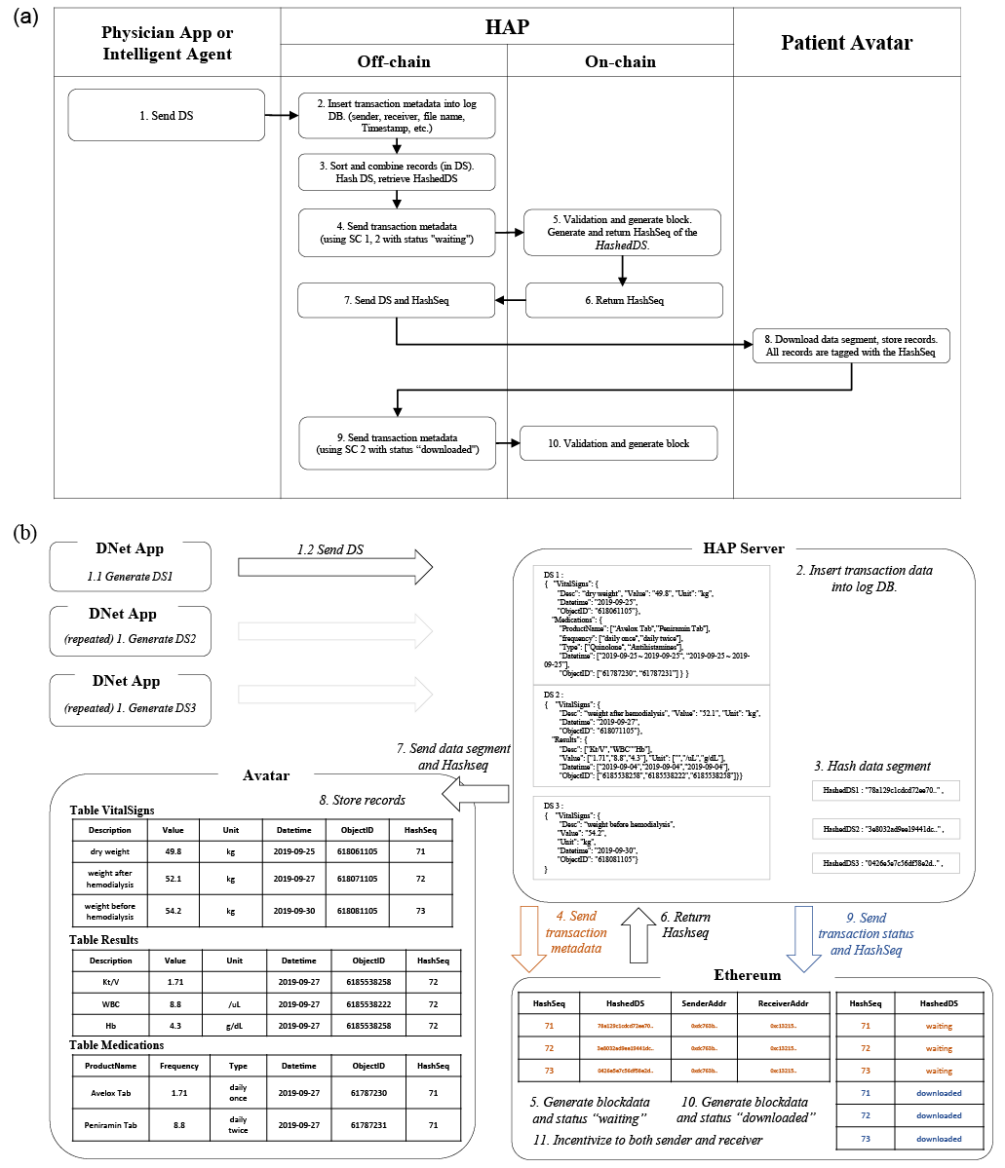
Process of transmitting health data from a physician App or third-party Agent to the patient Avatar. Health data transaction hash logs are generated and updated via smart contracts in Ethereum blockchain. Steps of three separate data transmissions from a physician App to the patient Avatar for PHR update are demonstrated as (a) a workflow diagram and (b) detailed illustration. SC : Smart Contract; DS : Data Segment; DB: database; DNet: DialysisNet; HAP: Health Avatar Platform; PHR: personal health record.

A data segment has one or more data elements with values (sample data sets can be found in Multimedia Appendix 3:Figures S1-S3). When a physician app initiates a data transmission of vital signs (or laboratory results or medications) to a patient Avatar, the HAP relay server saves the time-stamped logs of the sender, recipient, and file name, sorts the data elements in the data segment, and extracts *HashedDS* from the data segment via Secure Hash Algorithm-256 (Figure 2a). HAP then transfers the extracted *HashedDS* and blockchain account addresses of the sender and receiver to smart contract SC-1 health data transaction manager for execution (Figure 2b). The blockchain node creates transaction metadata as block data with SC-1 health data transaction manager executed. By executing SC-2 health data transaction status manager, block data are created and tagged with the “waiting” status for the relevant data transaction. If block creation is successful, a *HashSeq* value is created by the blockchain and SC-1 returns this *HashSeq*. *HashSeq* is a sequence number created by SC-1 health data transaction manager to serve as a unique identifier corresponding to the *HashedDS* value. Through SC-1 health data transaction manager, *HashedDS* is mapped to this *HashSeq* and stored in the blockchain. Because *HashSeq* can function as a foreign key in the 3 smart contracts, the metadata for the exchanged data segment can be managed by normalizing relative to each contract.

When *HashSeq* is returned from the blockchain, the patient Avatar is enabled by the server with a push message to receive the data segment and *HashSeq*. The patient Avatar stores all records included in the downloaded data segment file in the personal health record database. These records are tagged with *HashSeq* and updated in the personal health record (or the patient Avatar database). When the personal health record update process is completed, the patient Avatar transmits its status information to HAP, indicating that data downloading is complete. The HAP server then updates the status information of the data segment to “complete” through SC-2 health data transaction status manager to record the completion of the patient's health data transmission and the personal health record update on the blockchain. Until the data transmission process is completed, metadata pertaining to the 3 data segments transmitted are maintained in blockchain storage (Figure 2).

### Pull: Requesting and Receiving Data

**Figure 3:**
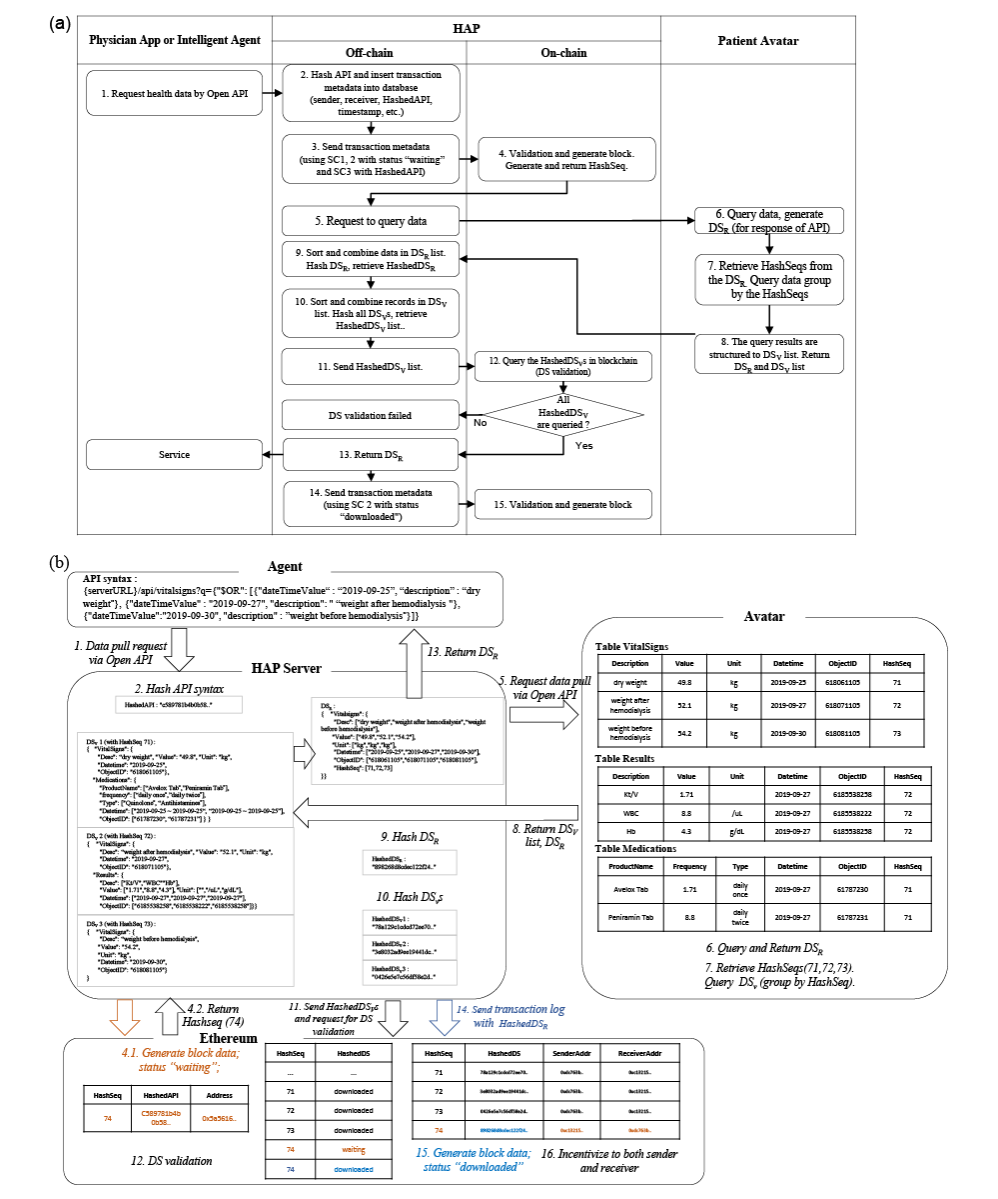
Process of requesting patient data and receiving data by an intelligent agent or a physician app: (a) workflow diagram and (b) detailed example of data flow initiated by an intelligent agent (or a physician app) requesting patient data stored in a patient Avatar for the purpose of providing clinical recommendations via Open API. DSV: data segment for validation; DSR: data segment for response; HAP: Health Avatar Platform; SC: smart contract.

The HAP server relays the request (Figure 3) through the proper API to the patient Avatar (Multimedia Appendix 4: Figure S4). After authorization and authentication, the Avatar responds to a proper and trustworthy request by returning 2 types of data segments: data segments for the response (DSR) and data segments for validation (DSV), as the query response for the API request and the query result for data segment validation, respectively. Data segment validation is a process that checks whether there is a modulated record in the data segment before returning the query result to the requester. An example transaction scenario (Figure 3) is an intelligent agent requesting a patient Avatar for personal health record data including “dry weight,” “weight after hemodialysis,” and “weight before hemodialysis” through open API complying with the HAP RESTful API syntax. The “/api/vitalSigns/” part of the API syntax refers to the database table *VitalSigns*, and the part next to *q* is the query string. The query string requests the dry weight measurement of “2019-09-25,” weight after hemodialysis of “2019-09-27,” and weight before hemodialysis of “2019-09-30.” After performing authentication and authorization for the requesting agent, HAP transmits the data request to the corresponding patient Avatar. In response to the request, the patient's Avatar queries 2 types of data segments: DSV and DSR. First, DSR is extracted from the patient Avatar’s personal health record database. Avatars' personal health record tables are devised on a data model that conforms to ASTM Continuity of Care Record and HL7 Continuity of Care Document standards. Health records previously delivered in the same data segment are tagged with the same *HashSeq* but are separately stored in 3 different tables (Figure 2b and Figure 3b) according to the data model. The corresponding data segments are pulled and processed to compile the query result for the requested API syntax and then returned to the requester.

Data segment validation, a process of verifying whether or not the transmitted DSR has been tampered with, is performed before the queried DSR is returned to the agent. A query using *HashSeq* included in the DSR is executed in the Avatar, resulting in a list of DSVs. Each DSV is bound to the *HashSeq* and regenerates *HashedDS* by sorting and hashing the records (or data elements) included in each DSV. If all regenerated *HashedDS*s are successfully retrieved from the blockchain, the DSV corresponding to the *HashedDS* has not been compromised. This also means that the records included in DSR have not been modified. Upon successful validation, information about the agent's data request by API is inserted into the block by the SC-3 HAP API transaction manager. This creates a block to update the status of the personal health record transaction to “complete” through the SC-2 health data transaction status manager. When transaction data generated by the agent API are created as block data, the server returns the DSR to the requesting agent. It was demonstrated that a smart contract in collaboration with data elements provided by metadata registry enable detailed data element–level query and access control beyond the resource-level query enabled by HL7 FHIR and other messaging standards. An authorized agent can provide highly personalized health care services without requesting an unnecessary amount of data beyond its declared capability and beyond what is authorization by HAP.

### Data Backup Process

**Figure 4:**
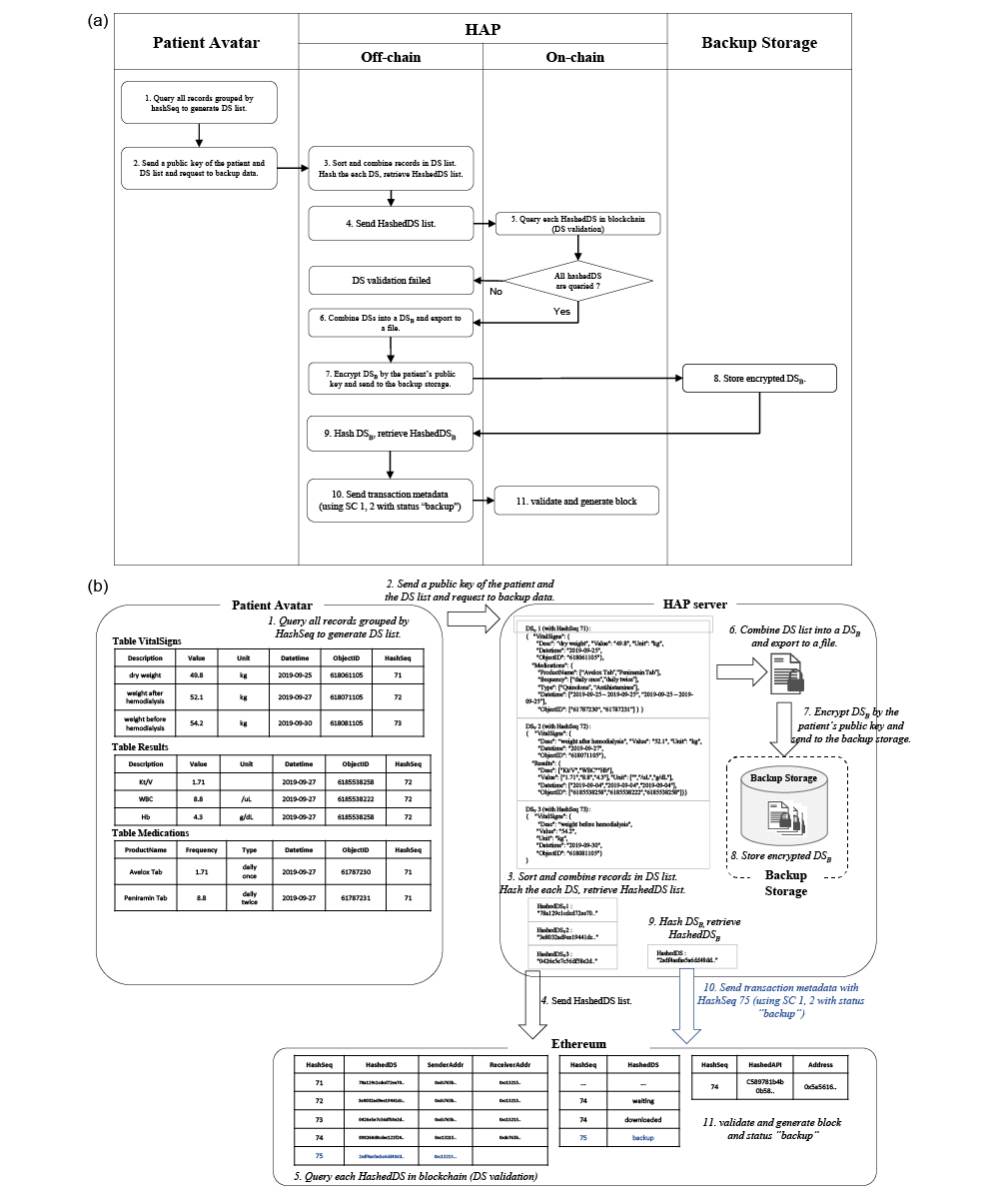
Data backup process: (a) workflow diagram and (b) use-case illustration initiated by an entity. DS: data segment; DSB: data segment for backup; HAP: Health Avatar Platform; SC: smart contract.

For the purpose of strong data privacy protection, HAP does not store any health data that are transmitted through the server; however, data backups are necessary for many purposes under strict patient control (Figure 4, Multimedia Appendix 4: Figure S5). When a patient initiates the backup process, the patient Avatar queries all personal health record data except for the patient identifier and transmits it to the HAP API with a backup request. The API allows one to output the data set grouped by *HashSeq*. Data segments transmitted through HAP will be hashed, and each *HashedDS* is created for validation. If validated passes, it means that the data segments to be backed up have not been altered and they are sent to the backup storage. Before the data segments are saved into backup storage, the verified segments are integrated into a data segment file. Using the patient's public key, the file is encrypted by the RSA (Rivest–Shamir–Adleman [40]) method and stored in the backup storage. When the encrypted file is stored in storage, the server creates *HashedDS* after hashing the integrated data segment of the file. To record the completion of the transaction in storage, the *HashedDS* “2sdf4asfas5a6dd48dd...” is connected to *HashSeq* 75 and stored. The blockchain stores the status of the transaction as “backup.”

## Discussion

### Principal Findings

The legacy HAP successfully performed decentralized health data management. From a data management perspective, decentralized management of personal health record with a patient's smartphone app is less efficient than a centralized approach; however, in terms of privacy protection and patient empowerment, decentralization is better for creating a highly interconnected mobile health ecosystem. We built a decentralized system and performed real-world clinical-practice validation with DialysisNet and RehabilitationNet. The platform successfully prevented data reuse and personal information leakages based on the trust of the system. The introduction of the blockchain and smart contracts significantly improved the efficiency and effectiveness of our decentralized health data management method. The adoption of blockchain to the legacy HAP system inevitably incurs overhead (Multimedia Appendix 5); however, we observed that the overhead, 32.58 ms on average, added to the legacy system was minimized by introducing asynchronous blockchain connection. The platform demonstrated that blockchain is a suitable software tool that safely and efficiently performs the required data verification and decentralized data backup processes.

HAP provides semantic interoperability for all data exchanges in the system. ASTM Continuity of Care Record and HL7 Continuity of Care Document standards were applied as a syntactic backbone required for HAP data management; however, syntactic standards alone are insufficient for a unified specification (eg, data type, format) for all data exchanges on the platform. Thus, we installed XNetHub in each health care institution (Figure 1) and metadata registry to ensure semantic interoperability among different electronic health records. HAP XNetHub supports HL7 FHIR, allowing resource-level health data queries. HAP treats a message (or a health record) as a collection of data segments composed of data elements, which are defined and managed by a metadata server in compliance with ISO/IEC 11179 metadata registry standards and provide thousands of expert curated common data elements required for hemodialysis patient management. One unique advantage of our work is that the blockchain-enabled HAP allows data segment–/data element–level querying and health data processing that are fully authenticated and audit trailed by the enabling technologies such as the immutable hash and sub–hash management schemes (Figures 2-4) with smart contracts (Table 2). In contrast, HL7 FHIR’s resource level data management does not allow granularity that is fine enough for health data querying or processing.

The introduction of metadata registry on top of these syntactic standards with predefined, preregistered, and postexpandable common data elements, highly enriched in semantics by means of standard vocabulary and ontology mappings, further improves semantic queries to each data element value level. Furthermore, we demonstrated that data segment– and data element–level data verifications were enabled by this architecture. A metadata registry improves the semantic interoperability of health data exchanges [8,41,42]. The semantic layer allows patients to integrate their health records from multiple health care institutions. Physicians can consolidate the health records of patients from different institutions. Moreover, third parties can have an integrated view of patients' health records through HAP RESTful APIs.

Blockchain and smart contract technologies were used in this platform to enhance the security of patient-centered personal health record transactions and the efficiency of decentralized data management. Additionally, for exchanges of patient data that may occur on the platform, HAP can provide incentives for data sharing to parties with whom the data are being exchanged. Many health care systems adopting fee-for-service reimbursement mechanism mainly reward highly materialized clinical services, such as medications, laboratory testing, or interventions, but lack sufficient reward systems for education, exercise, prevention, or long-term management that are more relevant for chronic conditions, which are ever increasing. Given all of these advantages, the HAP interconnected patient Avatars, physician apps, and intelligent agents system can become an ecosystem that promotes the reliable sharing of health data performed with patient empowerment.

### Limitations

Due to the features of the proof-of-authority consensus algorithm, a delay during block generation equal to the setting in the genesis block occurs; however, in this prototype system, the block data are generated through an asynchronous on-chain process apart from off-chain transactions for health data, meaning that there are no delays in off-chain data transactions. Another challenge arises when verifying the patient Avatar's personal health record data (through data backup or data query processes)—when a large message is exchanged, the speed of data verification and the return of the verification result may be slower. Accordingly, it may be necessary in the future to calculate the data alignment method included in the DSV and the appropriate time required during the process of hashing the data segment. For this process, a trade-off study on the time required for data processing and the size of the transmitted data segment may be required.

### Conclusions

We designed and built an ecosystem that provides efficient and effective decentralized health data management and exchange operations by applying a prototype blockchain and smart contract to a patient device–based personal health record system. It was demonstrated that health data access control and authenticity verification of personal health record data are enabled not only at the overall personal health record or resource level but also at granular data element and data value levels.

## Appendix

Multimedia Appendix 1 Transmitted data segment.

Multimedia Appendix 2 Health Avatar Platform participant service modules.

Multimedia Appendix 3 Comparison of blockchain-based health information systems.

Multimedia Appendix 4 Data segments with demo data sets.

Multimedia Appendix 5 Performance evaluation.

## Abbreviations

API: application programming interface
DSR: data segment for response
DSV: data segment for validation
FHIR: Fast Healthcare Interoperability Resources
HAP: Health Avatar Platform
IoT: Internet of Things
ISO/IEC: International Organization for Standardization/International Electrotechnical Commission
SC: smart contract
XNet: physician apps
XNetHub: institutional gateway server
