# Supplementary figures and images for: Smart Decentralization of Personal Health Records with Physician Apps and Helper Agents on Blockchain: Platform Design and Implementation Study
Source: JMIR Med Inform. 2021 Jun 7;9(6):e26230. doi: 10.2196/26230 (PMC8218219; doi:10.2196/26230)

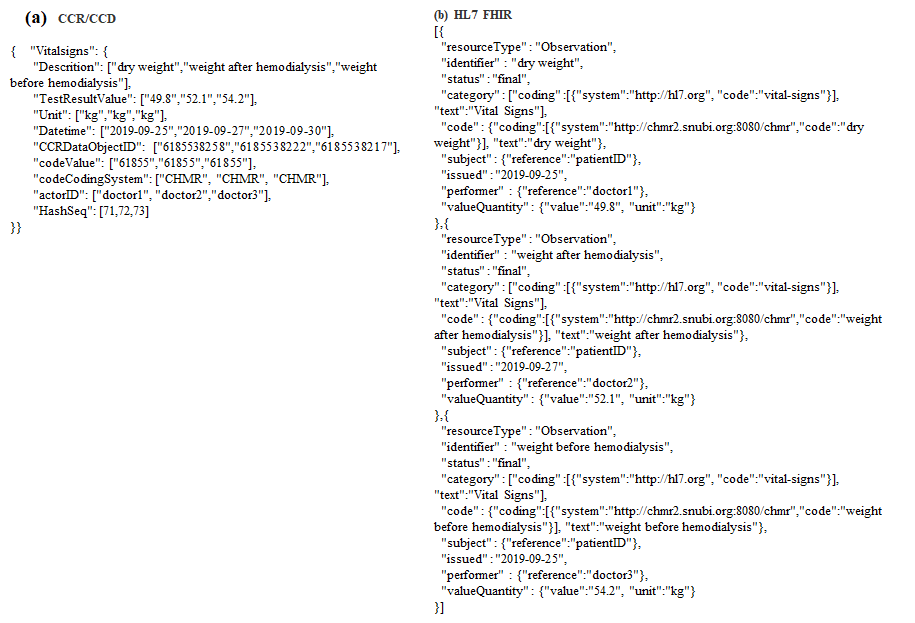

Supplement: Multimedia Appendix 1 [file medinform_v9i6e26230_app1.png]

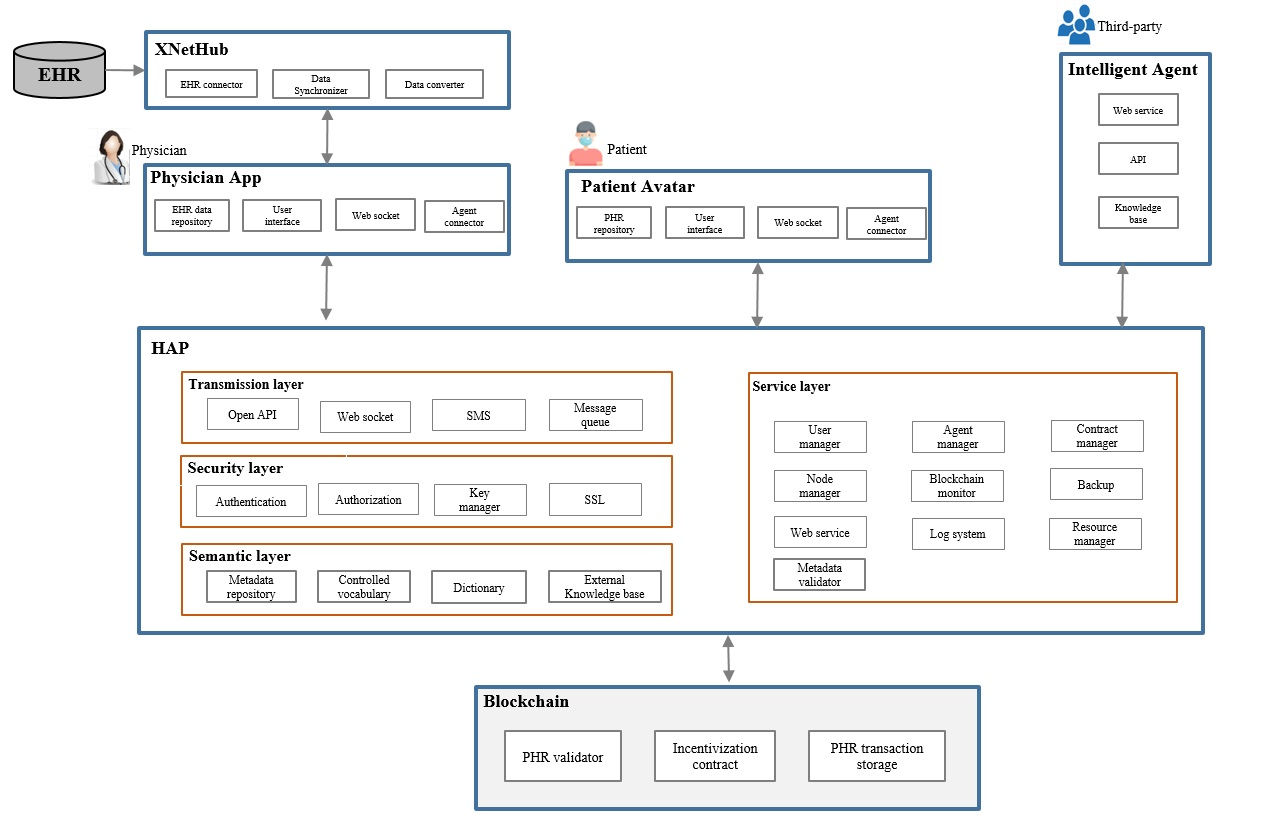

Supplement: Multimedia Appendix 2 [file medinform_v9i6e26230_app2.png]
