# Supplementary material for: Smart Decentralization of Personal Health Records with Physician Apps and Helper Agents on Blockchain: Platform Design and Implementation Study
Source: JMIR Med Inform. 2021 Jun 7;9(6):e26230. doi: 10.2196/26230 (PMC8218219; doi:10.2196/26230)
Supplement: Multimedia Appendix 3 [file medinform_v9i6e26230_app3.docx]

| **Author** | **EHR/PHR** | **Health data**  **location** | | | **Interoperability Standard** | | | | **Mode of data integration** | **Type of health data storage** | **PHR connectivity** | **Blockchain platform** |
| --- | --- | --- | --- | --- | --- | --- | --- | --- | --- | --- | --- | --- |
|  |  | **On-chain** | | **Off-chain** | | **Syntactic** | | **Semantic** |  |  |  |  |
| A Ekblaw et al. (MedREC) (2016) [16] | EHR | No | Provider system | | | | No | No | No integration | Decentralized | N/A | Ethereum |
| Dubovitskaya et al. (2020) [20] | EHR | No | Cloud | | | | FHIR | No | Integrated health systems | Centralized | N/A | Hyperledger Fabric |
| GG Dagher et al. (2018) [17] | EHR | No | Cloud | | | | No | No | Intermediary | Centralized | N/A | Ethereum |
| Motohashi et al. (2019) [21] | EHR | Yes | No | | | | No | No | Integrated health systems | Decentralized | N/A | Hyperledger Fabric |
| Anuraag A. Vazirani et al. (2020) [22] | EHR | No | Cloud | | | | No | No | Intermediary | Centralized | N/A | Self-development |
| Ray Hylock et al. (2019) [23] | PHR | Yes | No | | | | FHIR | No | Intermediary | Decentralized | Commercial-based PHR | Self-development |
| Roehrs et al. (2019) [24] | PHR | No | Overlay network nodes | | | | openEHR, FHIR | SNOMED-CT, LOINC, ICD, ISO | Intermediary | Distributed | Commercial-based PHR | Self-development |
| Ahmed Raza Rajput et al. (2019) [25] | PHR | Yes | No | | | | No | No | Integrated health systems | Decentralized | Commercial-based PHR | Hyperledger Fabric |
| Sandi Rahmadika et al. (2018) [29] | PHR | No | Overlay network nodes | | | | No | No | Intermediary | Peer-to-peer | Commercial-based PHR | Self-development |
| Thein Than Thwin et al. (2019) [26] | PHR | No | Cloud | | | | No | No | Integrated health systems | Centralized | Commercial-based PHR | Hyperledger Fabric |
| Shangping Wang et al. (2019) [27] | PHR | No | IPFS storage | | | | No | No | Integrated health systems | Distributed | Commercial-based PHR | Ethereum |
| Hsiu-An Lee et al. (2020) [28] | PHR | No | Cloud | | | | FHIR | No | Integrated health systems | Centralized | Provider-based PHR | Ethereum |
| Health Avatar | Both | No | Smart device with backup | | | | CCR/ CCD/ FHIR | ISO/IEC-11179, SNOMED-CT, UMLS, etc. | Intermediary + Patient-centered | Distributed | Patient’s device-based PHR | Ethereum |
