# Supplementary material for: Smart Decentralization of Personal Health Records with Physician Apps and Helper Agents on Blockchain: Platform Design and Implementation Study
Source: JMIR Med Inform. 2021 Jun 7;9(6):e26230. doi: 10.2196/26230 (PMC8218219; doi:10.2196/26230)
Supplement: Multimedia Appendix 4 [file medinform_v9i6e26230_app4.docx]

Multimedia Appendix 4. Data segments with demo-datasets

Figure S1. Data segment 1 standardized by HL7-CCD/CCR (represented in Figure 2 of main manuscript)

{

"syncCode": "insert",

"medications": [{

"prescriptionNumber": ["2019-09-25_dialysis", "2019-09-25_dialysis"],

"productName": ["Avelox Tab","Peniramin Tab"],

"codeValue": ["CHMR:61787-14925", "CHMR:61787-3215"],

"frequency": ["daily once","daily twice"],

"actorID": ["dialysis", "dialysis"],

"Type": ["Quinolone", "Antihistamines"],

"dateTimeValue": ["2019-09-25 ~ 2019-09-25", "2019-09-25 ~ 2019-09-25"],

"dateTimeType": ["prescriptionDate", "prescriptionDate"],

"codeCodingSystem": ["CHMR", "CHMR"],

"actorRole": ["physician","physician"],

"ObjectID": ["dialysis61787230", "dialysis61787231"]

}],

"vitalsigns": [{

"testResultValue": ["49.8"],

"codeValue": ["FSQID:61807", "FSQID:61808"],

"codeCodingSystem": ["CHMR", "CHMR"],

"actorID": ["dialysis", "dialysis"],

"dateTimeType": ["measured"],

"dateTimeValue": ["2019-09-25"],

"actorRole": ["physician"],

"description": ["dry weight"],

"testResultUnit": ["kg"],

"objectID": ["dialysis618061105"]

}]

}

Figure S2. Data segment 2 standardized by HL7-CCD/CCR (represented in Figure 2 of main manuscript)

{

"syncCode": "insert",

"vitalsigns": [{

"testResultValue": ["55.1", "52.1"],

"codeValue": ["FSQID:61807", "FSQID:61808"],

"codeCodingSystem": ["CHMR", "CHMR"],

"actorID": ["dialysis", "dialysis"],

"dateTimeType": ["measured", "measured"],

"dateTimeValue": ["2019-09-27", "2019-09-27"],

"actorRole": ["physician", "physician"],

"description": ["weight before hemodialysis", "weight after hemodialysis"],

"testResultUnit": ["kg", "kg"],

"objectID": ["dialysis618071105", "dialysis618081105"]

}],

"results": [{

"testResultValue": ["1.71", "8.8", "5.65", "2.41", "0.54", "0.18", "0.01", "217", "63.8", "13.6", "8.3", "140", "8.4", "5.8", "407.1", "7.5", "27", "154", "5.6", "3.7", "15", "10", "116", "132", "116", "37", "76", "0.57", "4.3", "Positive(49.55)", "Negative(0.043)"],

"codeValue": ["FSQID:61855-6", "FSQID:61855-9", "FSQID:61855-10", "FSQID:61855-11", "FSQID:61855-12", "FSQID:61855-13", "FSQID:61855-14", "FSQID:61855-15", "FSQID:61855-17", "FSQID:61855-18", "FSQID:61855-19", "FSQID:61855-20", "FSQID:61855-23", "FSQID:61855-24", "FSQID:61855-26", "FSQID:61855-27", "FSQID:61855-28", "FSQID:61855-30", "FSQID:61855-34", "FSQID:61855-35", "FSQID:61855-36", "FSQID:61855-37", "FSQID:61855-38", "FSQID:61855-39", "FSQID:61855-40", "FSQID:61855-41", "FSQID:61855-42", "FSQID:61855-43", "FSQID:61855-44", "FSQID:61855-45", "FSQID:61855-46"],

"codeCodingSystem": ["CHMR", "CHMR", "CHMR", "CHMR", "CHMR", "CHMR", "CHMR", "CHMR", "CHMR", "CHMR", "CHMR", "CHMR", "CHMR", "CHMR", "CHMR", "CHMR", "CHMR", "CHMR", "CHMR", "CHMR"],

"actorID": ["dialysis", "dialysis", "dialysis", "dialysis", "dialysis", "dialysis", "dialysis", "dialysis", "dialysis", "dialysis", "dialysis", "dialysis", "dialysis", "dialysis", "dialysis", "dialysis", "dialysis", "dialysis", "dialysis", "dialysis", "dialysis", "dialysis", "dialysis", "dialysis", "dialysis", "dialysis", "dialysis", "dialysis", "dialysis", "dialysis", "dialysis", "dialysis"],

"dateTimeType": ["measured", "measured", "measured", "measured", "measured", "measured", "measured", "measured", "measured", "measured", "measured", "measured", "measured", "measured", "measured", "measured", "measured", "measured", "measured", "measured", "measured", "measured", "measured", "measured", "measured", "measured", "measured", "measured", "measured", "measured", "measured"],

"dateTimeValue": ["2019-09-27", "2019-09-27", "2019-09-27", "2019-09-27", "2019-09-27", "2019-09-27", "2019-09-27", "2019-09-27", "2019-09-27", "2019-09-27", "2019-09-27", "2019-09-27", "2019-09-27", "2019-09-27", "2019-09-27", "2019-09-27", "2019-09-27", "2019-09-27", "2019-09-27", "2019-09-27", "2019-09-27", "2019-09-27", "2019-09-27", "2019-09-27", "2019-09-27", "2019-09-27", "2019-09-27", "2019-09-27", "2019-09-27", "2019-09-27", "2019-09-27", "2019-09-27"],

"actorRole": ["physician", "physician", "physician", "physician", "physician", "physician", "physician", "physician", "physician", "physician", "physician", "physician", "physician", "physician", "physician", "physician", "physician", "physician", "physician", "physician", "physician", "physician", "physician", "physician", "physician", "physician", "physician", "physician", "physician", "physician", "physician"],

"description": ["Kt/V", "WBC", "Neutrophil", "Lymph", "Monocyte", "Eosinophil", "Basophil", "Platelet", "PreBUN", "PostBUN", "Cr", "Na", "Ca", "P", "PTH", "Uric Acid", "Total CO2", "Glucose", "Protein", "Albumin", "AST", "ALT", "ALP", "T.Chol", "TG", "HDL", "LDL", "CRP", "Hb", "HBsAb", "HCV Ab"],

"type": ["blood test", "blood test", "blood test", "blood test", "blood test", "blood test", "blood test", "blood test", "blood test", "blood test", "blood test", "blood test", "blood test", "blood test", "blood test", "blood test", "blood test", "blood test", "blood test", "blood test", "blood test", "blood test", "blood test", "blood test", "blood test", "blood test", "blood test", "blood test", "blood test", "blood test", "blood test"],

"testResultUnit": ["", "\/uL", "\/uL", "\/uL", "\/uL", "\/uL", "\/uL", "\/uL", "mg\/dL", "mg\/dL", "mg\/dL", "mEq\/L", "mg\/dL", "mg\/dL", "pg\/mL", "mg\/dL", "mg\/dL", "mg\/dL", "g\/dL", "g\/dL", "IU\/L", "IU\/L", "IU\/L", "mg\/dL", "mg\/dL", "mg\/dL", "mg\/dL", "mg\/dL", "g\/dL", "", ""],

"objectID": ["dialysis6185538258", "dialysis6185538222", "dialysis6185538223", "dialysis6185538224", "dialysis6185538225", "dialysis6185538226", "dialysis6185538227", "dialysis6185538228", "dialysis6185538229", "dialysis6185538230", "dialysis6185538231", "dialysis6185538232", "dialysis6185538233", "dialysis6185538234", "dialysis6185538235", "dialysis6185538236", "dialysis6185538237", "dialysis6185538238", "dialysis6185538239", "dialysis6185538240", "dialysis6185538241", "dialysis6185538242", "dialysis6185538243", "dialysis6185538244", "dialysis6185538245", "dialysis6185538246", "dialysis6185538247", "dialysis6185538248", "dialysis6185538217", "dialysis6185538250", "dialysis6185538251"]

}]

}]

Figure S3. Data segment 3 standardized by HL7-CCD/CCR (represented in Figure 2 of main manuscript)

{

"syncCode": "insert",

"vitalsigns": [{

"testResultValue": ["54.2", "52.6"],

"codeValue": ["FSQID:61807", "FSQID:61808"],

"codeCodingSystem": ["CHMR", "CHMR"],

"actorID": ["dialysis", "dialysis"],

"dateTimeType": ["measured", "measured"],

"dateTimeValue": ["2019-09-30", "2019-09-30"],

"actorRole": ["physician", "physician"],

"description": ["weight before hemodialysis", "weight after hemodialysis"],

"testResultUnit": ["kg", "kg"],

"objectID": ["dialysis618071105", "dialysis618081105"]

}],

}

Figure S4. Data segment for data validation standardized by HL7-CCD/CCR (represented in Figure 3 of main manuscript)

[{

"syncCode": "insert",

"medications": [{

"prescriptionNumber": ["2019-09-25_dialysis", "2019-09-25_dialysis"],

"productName": ["Avelox Tab","Peniramin Tab"],

"codeValue": ["CHMR:61787-14925", "CHMR:61787-3215"],

"frequency": ["daily once","daily twice"],

"actorID": ["dialysis", "dialysis"],

"Type": ["Quinolone", "Antihistamines"],

"dateTimeValue": ["2019-09-25 ~ 2019-09-25", "2019-09-25 ~ 2019-09-25"],

"dateTimeType": ["prescriptionDate", "prescriptionDate"],

"codeCodingSystem": ["CHMR", "CHMR"],

"actorRole": ["physician","physician"],

"objectID": ["dialysis61787230", "dialysis61787231"],

"hashSeq": [71,71]

}],

"vitalsigns": [{

"testResultValue": ["49.8"],

"codeValue": ["FSQID:61806"],

"codeCodingSystem": ["CHMR"],

"actorID": ["dialysis"],

"dateTimeType": ["measured"],

"dateTimeValue": ["2019-09-25"],

"actorRole": ["physician"],

"description": ["dry weight"],

"testResultUnit": ["kg"],

"objectID": ["dialysis618061105"],

"hashSeq": [71]

}]

},

{

"syncCode": "insert",

"vitalsigns": [{

"testResultValue": ["55.1", "52.1"],

"codeValue": ["FSQID:61807", "FSQID:61808"],

"codeCodingSystem": ["CHMR", "CHMR"],

"actorID": ["dialysis", "dialysis"],

"dateTimeType": ["measured", "measured"],

"dateTimeValue": ["2019-09-27", "2019-09-27"],

"actorRole": [72, 72],

"description": ["weight before hemodialysis", "weight after hemodialysis"],

"testResultUnit": ["kg", "kg"],

"objectID": ["dialysis618071105", "dialysis618081105"],

"hashSeq": [72,72,72]

}],

"results": [{

"testResultValue": ["1.71", "8.8", "5.65", "2.41", "0.54", "0.18", "0.01", "217", "63.8", "13.6", "8.3", "140", "8.4", "5.8", "407.1", "7.5", "27", "154", "5.6", "3.7", "15", "10", "116", "132", "116", "37", "76", "0.57", "4.3", "Positive(49.55)", "Negative(0.043)"],

"codeValue": ["FSQID:61855-6", "FSQID:61855-9", "FSQID:61855-10", "FSQID:61855-11", "FSQID:61855-12", "FSQID:61855-13", "FSQID:61855-14", "FSQID:61855-15", "FSQID:61855-17", "FSQID:61855-18", "FSQID:61855-19", "FSQID:61855-20", "FSQID:61855-23", "FSQID:61855-24", "FSQID:61855-26", "FSQID:61855-27", "FSQID:61855-28", "FSQID:61855-30", "FSQID:61855-34", "FSQID:61855-35", "FSQID:61855-36", "FSQID:61855-37", "FSQID:61855-38", "FSQID:61855-39", "FSQID:61855-40", "FSQID:61855-41", "FSQID:61855-42", "FSQID:61855-43", "FSQID:61855-44", "FSQID:61855-45", "FSQID:61855-46"],

"codeCodingSystem": ["CHMR", "CHMR", "CHMR", "CHMR", "CHMR", "CHMR", "CHMR", "CHMR", "CHMR", "CHMR", "CHMR", "CHMR", "CHMR", "CHMR", "CHMR", "CHMR", "CHMR", "CHMR", "CHMR", "CHMR"],

"actorID": ["dialysis", "dialysis", "dialysis", "dialysis", "dialysis", "dialysis", "dialysis", "dialysis", "dialysis", "dialysis", "dialysis", "dialysis", "dialysis", "dialysis", "dialysis", "dialysis", "dialysis", "dialysis", "dialysis", "dialysis", "dialysis", "dialysis", "dialysis", "dialysis", "dialysis", "dialysis", "dialysis", "dialysis", "dialysis", "dialysis", "dialysis", "dialysis"],

"dateTimeType": ["measured", "measured", "measured", "measured", "measured", "measured", "measured", "measured", "measured", "measured", "measured", "measured", "measured", "measured", "measured", "measured", "measured", "measured", "measured", "measured", "measured", "measured", "measured", "measured", "measured", "measured", "measured", "measured", "measured", "measured", "measured"],

"dateTimeValue": ["2019-09-27", "2019-09-27", "2019-09-27", "2019-09-27", "2019-09-27", "2019-09-27", "2019-09-27", "2019-09-27", "2019-09-27", "2019-09-27", "2019-09-27", "2019-09-27", "2019-09-27", "2019-09-27", "2019-09-27", "2019-09-27", "2019-09-27", "2019-09-27", "2019-09-27", "2019-09-27", "2019-09-27", "2019-09-27", "2019-09-27", "2019-09-27", "2019-09-27", "2019-09-27", "2019-09-27", "2019-09-27", "2019-09-27", "2019-09-27", "2019-09-27", "2019-09-27"],

"actorRole": ["physician", "physician", "physician", "physician", "physician", "physician", "physician", "physician", "physician", "physician", "physician", "physician", "physician", "physician", "physician", "physician", "physician", "physician", "physician", "physician", "physician", "physician", "physician", "physician", "physician", "physician", "physician", "physician", "physician", "physician", "physician"],

"description": ["Kt/V", "WBC", "Neutrophil", "Lymph", "Monocyte", "Eosinophil", "Basophil", "Platelet", "PreBUN", "PostBUN", "Cr", "Na", "Ca", "P", "PTH", "Uric Acid", "Total CO2", "Glucose", "Protein", "Albumin", "AST", "ALT", "ALP", "T.Chol", "TG", "HDL", "LDL", "CRP", "Hb", "HBsAb", "HCV Ab"],

"type": ["blood test", "blood test", "blood test", "blood test", "blood test", "blood test", "blood test", "blood test", "blood test", "blood test", "blood test", "blood test", "blood test", "blood test", "blood test", "blood test", "blood test", "blood test", "blood test", "blood test", "blood test", "blood test", "blood test", "blood test", "blood test", "blood test", "blood test", "blood test", "blood test", "blood test", "blood test"],

"testResultUnit": ["", "\/uL", "\/uL", "\/uL", "\/uL", "\/uL", "\/uL", "\/uL", "mg\/dL", "mg\/dL", "mg\/dL", "mEq\/L", "mg\/dL", "mg\/dL", "pg\/mL", "mg\/dL", "mg\/dL", "mg\/dL", "g\/dL", "g\/dL", "IU\/L", "IU\/L", "IU\/L", "mg\/dL", "mg\/dL", "mg\/dL", "mg\/dL", "mg\/dL", "g\/dL", "", ""],

"objectID": ["dialysis6185538258", "dialysis6185538222", "dialysis6185538223", "dialysis6185538224", "dialysis6185538225", "dialysis6185538226", "dialysis6185538227", "dialysis6185538228", "dialysis6185538229", "dialysis6185538230", "dialysis6185538231", "dialysis6185538232", "dialysis6185538233", "dialysis6185538234", "dialysis6185538235", "dialysis6185538236", "dialysis6185538237", "dialysis6185538238", "dialysis6185538239", "dialysis6185538240", "dialysis6185538241", "dialysis6185538242", "dialysis6185538243", "dialysis6185538244", "dialysis6185538245", "dialysis6185538246", "dialysis6185538247", "dialysis6185538248", "dialysis6185538217", "dialysis6185538250", "dialysis6185538251"],

"hashSeq": [72, 72, 72, 72, 72, 72, 72, 72, 72, 72, 72, 72, 72, 72, 72, 72, 72, 72, 72, 72, 72, 72, 72, 72, 72, 72, 72, 72, 72, 72, 72]

}]

},

{

"syncCode": "insert",

"vitalsigns": [{

"testResultValue": ["54.2", "52.6"],

"codeValue": ["FSQID:61807", "FSQID:61808"],

"codeCodingSystem": ["CHMR", "CHMR"],

"actorID": ["dialysis", "dialysis"],

"dateTimeType": ["measured", "measured"],

"dateTimeValue": ["2019-09-30", "2019-09-30"],

"actorRole": ["physician", "physician"],

"description": ["weight before hemodialysis", "weight after hemodialysis"],

"testResultUnit": ["kg", "kg"],

"objectID": ["dialysis618071105", "dialysis618081105"],

"hashSeq": [73,73]

}],

}]

Figure S5. Data segment for data backup standardized by HL7-CCD/CCR (represented in Figure 4 of main manuscript)

{

"syncCode": "insert",

"vitalsigns": [{

"testResultValue": ["49.8","52.1","54.2"],

"codeValue": ["FSQID:61806", "FSQID:61807","FSQID:61808"],

"codeCodingSystem": ["CHMR", "CHMR"],

"actorID": ["dialysis", "dialysis"],

"dateTimeType": ["measured","measured","measured"],

"dateTimeValue": ["2019-09-25","2019-09-27","2019-09-30"],

"actorRole": ["physician", "physician"],

"description": ["dry weight","weight after hemodialysis","weight before hemodialysis"],

"testResultUnit": ["kg","kg","kg"],

"objectID": ["dialysis618061105","dialysis618071105","dialysis618081105"],

"hashSeq": [71,72,73]

}],

}

{ "Vitalsigns": {

"Desc": ["dry weight","weight after hemodialysis","weight before hemodialysis"],

"Value": ["49.8","52.1","54.2"],

"Unit": ["kg","kg","kg"],

"Datetime": ["2019-09-25","2019-09-27","2019-09-30"],

"ObjectID": ["6185538258","6185538222","6185538217"],

"HashSeq": [71,72,73]

}}

Data segment for backup

[{

"syncCode": "insert",

"medications": [{

"prescriptionNumber": ["2019-09-25_dialysis", "2019-09-25_dialysis"],

"productName": ["Avelox Tab","Peniramin Tab"],

"codeValue": ["CHMR:61787-14925", "CHMR:61787-3215"],

"frequency": ["daily once","daily twice"],

"actorID": ["dialysis", "dialysis"],

"Type": ["Quinolone", "Antihistamines"],

"dateTimeValue": ["2019-09-25 ~ 2019-09-25", "2019-09-25 ~ 2019-09-25"],

"dateTimeType": ["prescriptionDate", "prescriptionDate"],

"codeCodingSystem": ["CHMR", "CHMR"],

"actorRole": ["physician","physician"],

"objectID": ["dialysis61787230", "dialysis61787231"],

"hashSeq": [71,71]

}],

"vitalsigns": [{

"testResultValue": ["49.8"],

"codeValue": ["FSQID:61806"],

"codeCodingSystem": ["CHMR"],

"actorID": ["dialysis"],

"dateTimeType": ["measured"],

"dateTimeValue": ["2019-09-25"],

"actorRole": ["physician"],

"description": ["dry weight"],

"testResultUnit": ["kg"],

"objectID": ["dialysis618061105"],

"hashSeq": [71]

}]

},

{

"syncCode": "insert",

"vitalsigns": [{

"testResultValue": ["55.1", "52.1"],

"codeValue": ["FSQID:61807", "FSQID:61808"],

"codeCodingSystem": ["CHMR", "CHMR"],

"actorID": ["dialysis", "dialysis"],

"dateTimeType": ["measured", "measured"],

"dateTimeValue": ["2019-09-27", "2019-09-27"],

"actorRole": [72, 72],

"description": ["weight before hemodialysis", "weight after hemodialysis"],

"testResultUnit": ["kg", "kg"],

"objectID": ["dialysis618071105", "dialysis618081105"],

"hashSeq": [72,72,72]

}],

"results": [{

"testResultValue": ["1.71", "8.8", "5.65", "2.41", "0.54", "0.18", "0.01", "217", "63.8", "13.6", "8.3", "140", "8.4", "5.8", "407.1", "7.5", "27", "154", "5.6", "3.7", "15", "10", "116", "132", "116", "37", "76", "0.57", "4.3", "Positive(49.55)", "Negative(0.043)"],

"codeValue": ["FSQID:61855-6", "FSQID:61855-9", "FSQID:61855-10", "FSQID:61855-11", "FSQID:61855-12", "FSQID:61855-13", "FSQID:61855-14", "FSQID:61855-15", "FSQID:61855-17", "FSQID:61855-18", "FSQID:61855-19", "FSQID:61855-20", "FSQID:61855-23", "FSQID:61855-24", "FSQID:61855-26", "FSQID:61855-27", "FSQID:61855-28", "FSQID:61855-30", "FSQID:61855-34", "FSQID:61855-35", "FSQID:61855-36", "FSQID:61855-37", "FSQID:61855-38", "FSQID:61855-39", "FSQID:61855-40", "FSQID:61855-41", "FSQID:61855-42", "FSQID:61855-43", "FSQID:61855-44", "FSQID:61855-45", "FSQID:61855-46"],

"codeCodingSystem": ["CHMR", "CHMR", "CHMR", "CHMR", "CHMR", "CHMR", "CHMR", "CHMR", "CHMR", "CHMR", "CHMR", "CHMR", "CHMR", "CHMR", "CHMR", "CHMR", "CHMR", "CHMR", "CHMR", "CHMR"],

"actorID": ["dialysis", "dialysis", "dialysis", "dialysis", "dialysis", "dialysis", "dialysis", "dialysis", "dialysis", "dialysis", "dialysis", "dialysis", "dialysis", "dialysis", "dialysis", "dialysis", "dialysis", "dialysis", "dialysis", "dialysis", "dialysis", "dialysis", "dialysis", "dialysis", "dialysis", "dialysis", "dialysis", "dialysis", "dialysis", "dialysis", "dialysis", "dialysis"],

"dateTimeType": ["measured", "measured", "measured", "measured", "measured", "measured", "measured", "measured", "measured", "measured", "measured", "measured", "measured", "measured", "measured", "measured", "measured", "measured", "measured", "measured", "measured", "measured", "measured", "measured", "measured", "measured", "measured", "measured", "measured", "measured", "measured"],

"dateTimeValue": ["2019-09-27", "2019-09-27", "2019-09-27", "2019-09-27", "2019-09-27", "2019-09-27", "2019-09-27", "2019-09-27", "2019-09-27", "2019-09-27", "2019-09-27", "2019-09-27", "2019-09-27", "2019-09-27", "2019-09-27", "2019-09-27", "2019-09-27", "2019-09-27", "2019-09-27", "2019-09-27", "2019-09-27", "2019-09-27", "2019-09-27", "2019-09-27", "2019-09-27", "2019-09-27", "2019-09-27", "2019-09-27", "2019-09-27", "2019-09-27", "2019-09-27", "2019-09-27"],

"actorRole": ["physician", "physician", "physician", "physician", "physician", "physician", "physician", "physician", "physician", "physician", "physician", "physician", "physician", "physician", "physician", "physician", "physician", "physician", "physician", "physician", "physician", "physician", "physician", "physician", "physician", "physician", "physician", "physician", "physician", "physician", "physician"],

"description": ["Kt/V", "WBC", "Neutrophil", "Lymph", "Monocyte", "Eosinophil", "Basophil", "Platelet", "PreBUN", "PostBUN", "Cr", "Na", "Ca", "P", "PTH", "Uric Acid", "Total CO2", "Glucose", "Protein", "Albumin", "AST", "ALT", "ALP", "T.Chol", "TG", "HDL", "LDL", "CRP", "Hb", "HBsAb", "HCV Ab"],

"type": ["blood test", "blood test", "blood test", "blood test", "blood test", "blood test", "blood test", "blood test", "blood test", "blood test", "blood test", "blood test", "blood test", "blood test", "blood test", "blood test", "blood test", "blood test", "blood test", "blood test", "blood test", "blood test", "blood test", "blood test", "blood test", "blood test", "blood test", "blood test", "blood test", "blood test", "blood test"],

"testResultUnit": ["", "\/uL", "\/uL", "\/uL", "\/uL", "\/uL", "\/uL", "\/uL", "mg\/dL", "mg\/dL", "mg\/dL", "mEq\/L", "mg\/dL", "mg\/dL", "pg\/mL", "mg\/dL", "mg\/dL", "mg\/dL", "g\/dL", "g\/dL", "IU\/L", "IU\/L", "IU\/L", "mg\/dL", "mg\/dL", "mg\/dL", "mg\/dL", "mg\/dL", "g\/dL", "", ""],

"objectID": ["dialysis6185538258", "dialysis6185538222", "dialysis6185538223", "dialysis6185538224", "dialysis6185538225", "dialysis6185538226", "dialysis6185538227", "dialysis6185538228", "dialysis6185538229", "dialysis6185538230", "dialysis6185538231", "dialysis6185538232", "dialysis6185538233", "dialysis6185538234", "dialysis6185538235", "dialysis6185538236", "dialysis6185538237", "dialysis6185538238", "dialysis6185538239", "dialysis6185538240", "dialysis6185538241", "dialysis6185538242", "dialysis6185538243", "dialysis6185538244", "dialysis6185538245", "dialysis6185538246", "dialysis6185538247", "dialysis6185538248", "dialysis6185538217", "dialysis6185538250", "dialysis6185538251"],

"hashSeq": [72, 72, 72, 72, 72, 72, 72, 72, 72, 72, 72, 72, 72, 72, 72, 72, 72, 72, 72, 72, 72, 72, 72, 72, 72, 72, 72, 72, 72, 72, 72]

}]

},

{

"syncCode": "insert",

"vitalsigns": [{

"testResultValue": ["54.2", "52.6"],

"codeValue": ["FSQID:61807", "FSQID:61808"],

"codeCodingSystem": ["CHMR", "CHMR"],

"actorID": ["dialysis", "dialysis"],

"dateTimeType": ["measured", "measured"],

"dateTimeValue": ["2019-09-30", "2019-09-30"],

"actorRole": ["physician", "physician"],

"description": ["weight before hemodialysis", "weight after hemodialysis"],

"testResultUnit": ["kg", "kg"],

"objectID": ["dialysis618071105", "dialysis618081105"],

"hashSeq": [73,73]

}],

}]
