# Supplementary material for: Smart Decentralization of Personal Health Records with Physician Apps and Helper Agents on Blockchain: Platform Design and Implementation Study
Source: JMIR Med Inform. 2021 Jun 7;9(6):e26230. doi: 10.2196/26230 (PMC8218219; doi:10.2196/26230)
Supplement: Multimedia Appendix 5 [file medinform_v9i6e26230_app5.docx]

**Multimedia Appendix 5.** Performance evaluation

#### *Study Design*

We designed a simulation test to measure the time costs of transaction management in blockchain. In order to measure the time costs of the blockchain, we compared time consumption of both systems, the legacy HAP and the blockchain applied HAP. In particular, in order to compare the performances between synchronous and asynchronous adoption of blockchain in HAP, the time consumption was independently measured in these two modes of the blockchain system. We defined the synchronous blockchain mode as a state in which the process flow of off-chain is delayed until block generation in on-chain process is completed, also the asynchronous blockchain mode is defined as a state in which the process flow of the off-chain is not delayed for block generation in on-chain process.

The time consumptions were measured on HAP servers, excluding network connection time or latency in terminal devices and applications. Time consumption was measured in the processes of data transmission to the patient (data push) and data transmission from the patient (data pull). 5 demo-datasets of a virtual hemodialysis patient were set for the test. This datasets contain common data elements of vital sign records such as dry weight, pre-dialysis weight, post-dialysis weight, height, etc. The datasets are organized according to the number of data elements (the number of date elements: 150, 300, 450, 600 and 750).

#### *Result of Evaluation*

The simulation test was performed to compare the performance between the legacy HAP system and the blockchain-applied HAP system. The 5 demo-datasets used in this simulation were classified according to the number of data elements for a virtual patient. Using these datasets, we performed data push and pull processes on each system and measured the processing time for each process. The time consumptions measured in HAP server, and the network connection time or latency with terminal devices, applications, and blockchain was excluded. Multimedia Appendix 5: Figure S1 (a) and Multimedia Appendix 5: Figure S1 (b) show the result of measured time consumption in legacy system and HAP with asynchronous blockchain mode. Multimedia Appendix 5: Figure S1(a) is time consumption on data push process from a physician app to a patient Avatar, Multimedia Appendix 5: Figure S1 (b) is time consumption on data pull process by an Agent app from a patient. As can be shown in Multimedia Appendix 5: Figure S1 (a) and Multimedia Appendix 5: Figure S1 (b), the time consumption increased in proportion to the number of transmitted data elements, and this phenomenon occurred identically in both systems, legacy HAP and HAP with asynchronous blockchain.


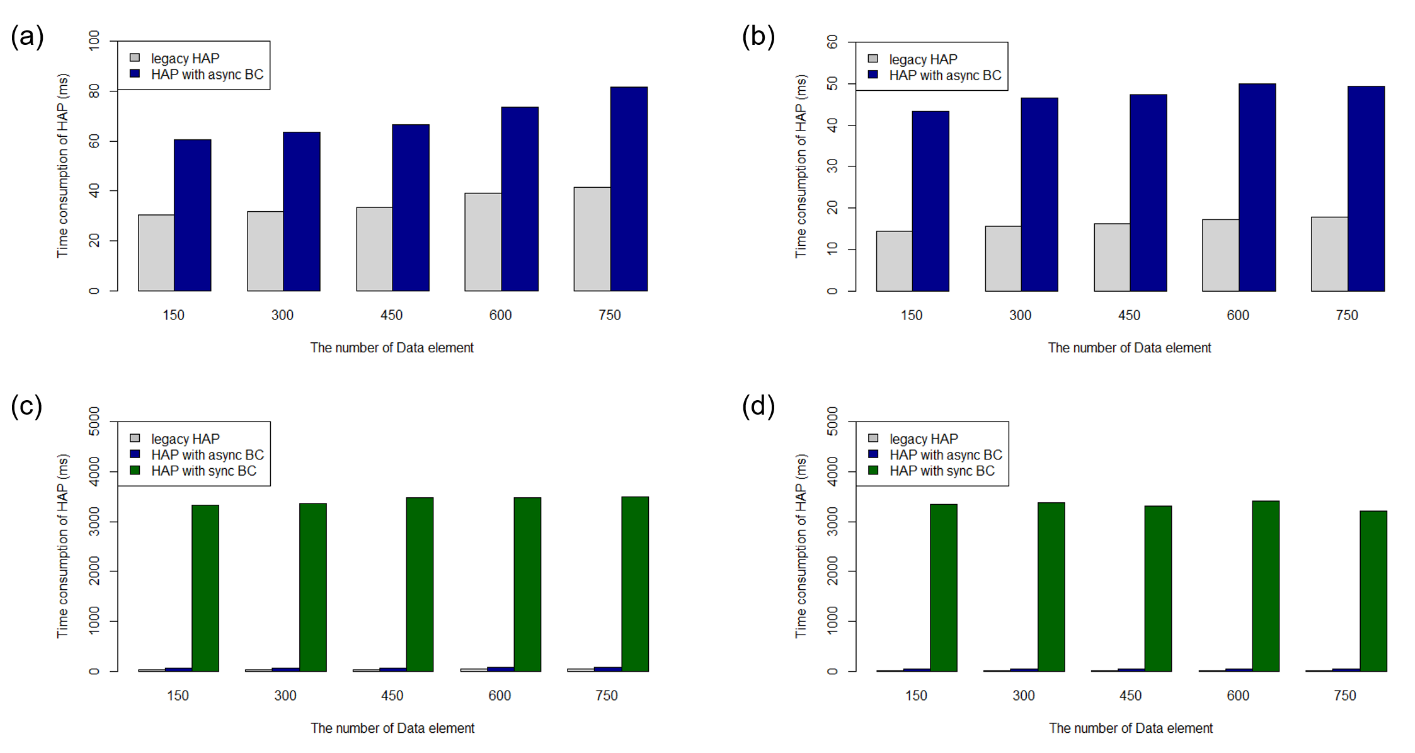


Figure S1. Measured time consumptions on data transmission executed in the legacy HAP system and the HAP system with two blockchain modes (asynchronized and synchronized blockchain). (a), (c) data push process: a physician app to a patient Avatar. (b), (d) data pull process: by an Agent app from a patient Avatar.

The processing time in the HAP system with the asynchronous blockchain takes 32.58ms more on average than the legacy HAP system. However, as shown in Multimedia Appendix 5: Figure S1 (c) and (d), it can be seen that the transaction processing time that occurred in the synchronous blockchain mode took 3378.92ms on average more than the legacy system. This overhead was caused by delaying the off-chain process until the generation of block data in the on-chain processes was completed.

In summary, as expected, the adoption of blockchain to the legacy system inevitably incurs overhead, but the delicate overhead generated through adoption of asynchronous blockchain does not affect data communications.
